# Virtual street crossing and scanning behavior in people with hemianopia: A step toward successful crossings

**DOI:** 10.1167/jov.25.11.1

**Published:** 2025-09-02

**Authors:** Eva M. J. L. Postuma, Gera A. de Haan, Joost Heutink, Frans W. Cornelissen

**Affiliations:** 1 Department Clinical and Developmental Neuropsychology, Faculty of Behavioral and Social Sciences, Rijksuniversiteit Groningen, Groningen, The Netherlands; 2 Royal Dutch Visio, Centre of Expertise for Blind and Partially Sighted People, Huizen, The Netherlands; 3 Laboratory for Experimental Ophthalmology, University Medical Center Groningen, University of Groningen, Groningen, The Netherlands

**Keywords:** hemianopia, street crossing, rehabilitation, scanning behavior, virtual reality

## Abstract

Individuals with homonymous hemianopia (HH) may benefit from adopting compensatory crossing and scanning strategies to successfully cross streets. In this study, we explored the effect of HH on street crossing outcomes, crossing behavior and scanning behavior in a virtual environment. Individuals with real HH (*N* = 18), unimpaired vision (*N* = 18), and simulated HH (*N* = 18) crossed a virtual street displayed through a head-mounted display. Virtual cars approached from both directions, traveling at a speed of either 30 or 50 km/h. Participants' crossing and scanning behaviors were recorded and analyzed across groups and the two car speeds. Although individuals with real and simulated HH took more time to cross compared to individuals with unimpaired vision depending on the car speed, the number of collisions and time-to-contact after crossings did not differ between groups. We observed no differences in the selection of car gaps, crossing initiation, and scanning behavior between groups. Our findings suggest that individuals with real and simulated HH align their crossing behavior to their visuomotor capabilities by using varying compensatory strategies. HH did not alter scanning behavior before crossing a virtual street. Despite its current shortcomings, virtual reality holds promise for street crossing research and rehabilitation.

## Introduction

The ability to successfully cross a street is an essential aspect of safe mobility. For individuals with a vision impairment such as homonymous hemianopia (HH), this can be particularly challenging. In this article, the term HH refers to vision loss in one half of the visual field because of acquired post-chiasmatic brain injury, including quadrantanopia. Consequently, in a single glance, individuals with HH can only perceive a portion of the visual information that those with unimpaired vision can perceive. Indeed, individuals with HH are known to encounter challenges in detecting road users during driving (Bowers, Ananyev, Mandel, Goldstein, & Peli, 2014; Papageorgiou, Hardiess, Mallot, & Schiefer, 2012; Swan, Savage, Zhang, & Bowers, 2021; Tant, Brouwer, Cornelissen, & Kooijman, 2002a), as well as detecting moving objects while walking (Iorizzo, Riley, Hayhoe, & Huxlin, 2011). Despite these concerns, how HH impacts pedestrian street crossing is not well understood.

To successfully cross a street, it is crucial that one's crossing behavior aligns with one's visuomotor capabilities. For instance, older adults may require more time to cross, reducing the time between crossing completion and a potential collision with an oncoming car (Lobjois & Cavallo, 2009; Pala et al., 2021). This latter metric, defined as the time-to-contact after a crossing, is often used to assess crossing outcomes. Selecting longer gaps between cars could mitigate the effect of longer crossing times on the time-to-contact. Yet, older adults tend to select shorter car gaps when the cars are traveling faster (Lobjois & Cavallo, 2009; Pala et al., 2021). Conversely, they seem to use a compensatory strategy of initiating their crossing more quickly, which could potentially maintain crossing outcomes by increasing the time-to-contact. Whether individuals with HH also use such compensatory crossing strategies to improve their crossing outcomes is currently unknown. Therefore, to thoroughly understand how individuals with HH manage to successfully cross streets, it is necessary to study their crossing behavior in detail.

Besides crossing behavior, scanning behavior could be a determinant of successful crossings in individuals with HH, as it contributes to successful task completion in various other mobility tasks (Bahnemann et al., 2015; Kasneci, Sippel, Aehling, et al., 2014; Kasneci, Sippel, Heister, et al., 2014; Kubler et al., 2015; Papageorgiou et al., 2012; Postuma et al., 2024; Swan et al., 2021). For instance, individuals with HH who employed adequate compensatory scanning exhibited fewer collisions with other road-users while crossing intersections in a driving simulator (Papageorgiou et al., 2012; Swan et al., 2021). Their scanning strategy involved fixating more frequently on task-relevant objects, making longer eye- and head movements when approaching a crossing, and having an extended scanning span. Additionally, exploration by making head movements has been proposed as a critical scanning characteristic for successful crossings in individuals with visual impairments such as HH, particularly in the moment leading up to the decision-to-cross (Hassan, Geruschat, & Turano, 2005; Pundlik et al., 2023). Last, during real-life streets crossing, individuals with HH more frequently perform gaze scans towards their blind hemispace and move their gaze slightly further into their blind hemispace compared to into their visible hemispace (approximately 36° vs. 40°), suggesting they use compensatory scanning (Pundlik et al., 2023).

Gaining more insight into compensatory crossing and scanning strategies is essential for optimizing the street crossing outcomes of individuals with HH. The obtained knowledge may enhance the mobility-related rehabilitation of individuals with HH (De Haan, Melis-Dankers, Brouwer, Tucha, & Heutink, 2015; De Haan, Melis-Dankers, Brouwer, Tucha, & Heutink, 2016), because it informs on compensatory strategies that individuals with HH could effectively utilize to alter their street crossing outcomes.

Yet, studying both crossing outcomes and compensatory behavior during street crossing poses a challenge. Participants should cross streets in a sufficiently challenging, yet safe environment, while minimizing the impact of uncontrolled environmental factors. However, street crossing in real-world scenarios is inherently unstandardized, unpredictable and uncontrollable. In contrast, virtual street crossing environments may serve a promising role by offering a safe, standardized and controllable means of evaluating street crossing and scanning behavior. It also offers the opportunity to simulate the presence of a visual field defect in visually unimpaired individuals (Biebl et al., 2022; Jones, Somoskeöy, Chow-Wing-Bom, & Crabb, 2020; Neugebauer et al., 2024; Wu, Ashmead, Adams, & Bodenheimer, 2018). Such simulations do not fully replicate the experience of individuals with HH. Within a simulation, individuals observe the vision loss, while those with actual HH may not even be fully aware of their impairment. Still, simulations can still offer insights into alterations in scanning behavior which arise from loss of vision, independent of any comorbidities that may be associated with the acquired brain injury (Biebl et al., 2022; Tant et al., 2002b). Additionally, they can provide insights into the compensatory strategies employed during the acute stage of vision loss (Biebl et al., 2022). While virtual street crossing environments seem promising, they are not without limitations. For instance, visually unimpaired individuals seem to select more unsafe crossing gaps in virtual reality compared to real-life, especially at faster car speeds. This is possibly due to difficulties with accurately detecting virtual car speed (Feldstein & Dyszak, 2020; Schneider et al., 2022). Nevertheless, in our view, virtual reality provides unique opportunities to study scanning and crossing behavior in ways not feasible in real-life, underscoring its value for research.

In this study, we aim to explore the effect of HH on street crossing outcomes, crossing behavior and scanning behavior by presenting a virtual environment by means of a head mounted display. We examined whether people with either real or simulated HH show different crossing outcomes, crossing behavior and scanning behavior while crossing virtual streets compared to individuals with unimpaired vision. The findings of this study may shed light on how HH affects street crossing and the compensatory strategies individuals with HH may employ. By including participants with simulated HH, this study may provide insights on whether these effects and compensatory strategies primarily stem from the visual field defect rather than comorbidities of the acquired brain injury (Biebl, Arcidiacono, Kacianka, Rieger, & Bengler, 2022; Tant, Cornelissen, Kooijman, & Brouwer, 2002b), and may be present during the acute stage of HH (Biebl et al., 2022). Overall, this study represents an initial step toward identifying the compensatory strategies that contribute to successful street crossing in individuals with HH.

## Methods

### Participants

A total of 58 individuals participated in this study, comprising 18 individuals with real HH, 20 individuals with unimpaired vision (including two drop-outs), and 18 individuals with simulated HH. Participants with HH were recruited via Royal Dutch Visio's rehabilitation centers in the Netherlands. Age- and gender-matched participants in the unimpaired vision and simulated HH groups were recruited via social media advertisements. The inclusion criteria for all participants were: age ≥ 18 years old, self-reported normal or corrected to normal vision, and a Mini-Mental State Examination score of 24 or higher (Folstein, Folstein, & McHugh, 1975). For the following inclusion criteria, individuals with HH underwent a screening process as part of their regular intake at the Royal Dutch Vision, while individuals with unimpaired vision provided self-reported data through a questionnaire. These criteria were no impairments in eye- or head-motility, no psychiatric disorders, no severe cognitive impairments, no impairments of balance or orientation, no language or verbal communication impairments, and no (other) visual or neurological disorders, such as neglect. After inclusion, the clock drawing test was performed at the start of the experiment as an additional check for symptoms of neglect. Additional inclusion criteria specific to the HH group included: having a homonymous visual field defect of at least quadrantanopia level with a neurological cause, no visual field defect on the ipsilesional side, a time since onset of ≥ 3 months, and a binocular visual acuity of Snellen ≥ 0.5 (6/12 or 20/40, logMAR 0.3). Two participants with unimpaired vision dropped out during the experiment because of technical problems and balance issues, respectively. These participants were replaced by two new participants. The demographics of the participants included for analysis are detailed in Table 1. The study received approval from the medical ethical committee of the University Medical Center Groningen (NL72491.042.20) and all participants gave informed consent.

**Table 1.** Demographic information of the three participant groups. *Notes*: HH = participants with real homonymous hemianopia; UN = participants with unimpaired vision; SH = participants with simulated homonymous hemianopia. *Asterisk* indicates compensatory scanning training of Royal Dutch Visio (De Haan et al., 2015, De Haan et al., 2016), Stage 0: training did not start, Stage 1: practicing scanning while walking inside, Stage 2: practicing scanning while walking outside, Stage 3: practicing scanning while cycling outside. *Dagger* indicates no training was necessary according to the occupational therapist.

Participant group (N)	HH (18)	UN (18)	SH (18)
Age in years (mean (SD) [range])	62 (18) [21–86]	61 (17) [22–79]	61 (17) [21–82]
Gender (male)	83.3% (15)	83.3% (15)	83.3% (15)
Cause of HH			
Stroke	77.8% (14)	—	—
Traumatic brain injury	16.6% (3)	—	—
Tumor	5.6% (1)	—	—
Side of HH (right)	44.4% (8)	—	44.4% (8)
Type of HH			
Hemianopia	61.1% (11)		61.1% (11)
Quadrantanopia	38.9% (7)	—	38.9% (7)
Macular sparing > 5° (Yes in % (N))	38.9% (7)	—	38.9% (7)
Time since onset in months, mean (SD) [range])	20.1 (12.8) [7–49]	—	—
Completion compensatory scanning training^*^			
Stage 0	11.1% (2)	—	—
Stage 1	11.1% (2)	—	—
Stage 2	5.6% (1)	—	—
Stage 3	5.6% (1)	—	—
Finished	38.9% (7)	—	—
No training necessary^†^	27.8% (5)	—	—

### Apparatus

The virtual street crossing environment was displayed by the HTC Vive Pro Eye (HTC Corporation, Taoyuan, Taiwan). This head mounted display together with two HTC Vive Base Stations (HTC Corporation, Taoyuan, Taiwan) was used to collect data on head rotation and position. This head-mounted display has a display resolution of 1440 (v) × 1600 (h) pixels for each eye. The horizontal and vertical fields of view were approximately 90° and 80°, respectively. Head motion and rotation was tracked by the HTC Vive base stations 2.0 (HTC Corporation). The integrated eye tracker (Tobii XR) recorded the eyes’ positions at 90 Hz. The eye-tracker requires a five-point calibration and has an accuracy of 0.5-1.1 degrees according to the manufacturer. The eye-tracking data was accessed through the Vive SRanpial SDK (HTC Corporation), providing filtered data that prevents gaze trembling when looking at distant objects and reduces precision error.

**Figure 1.**
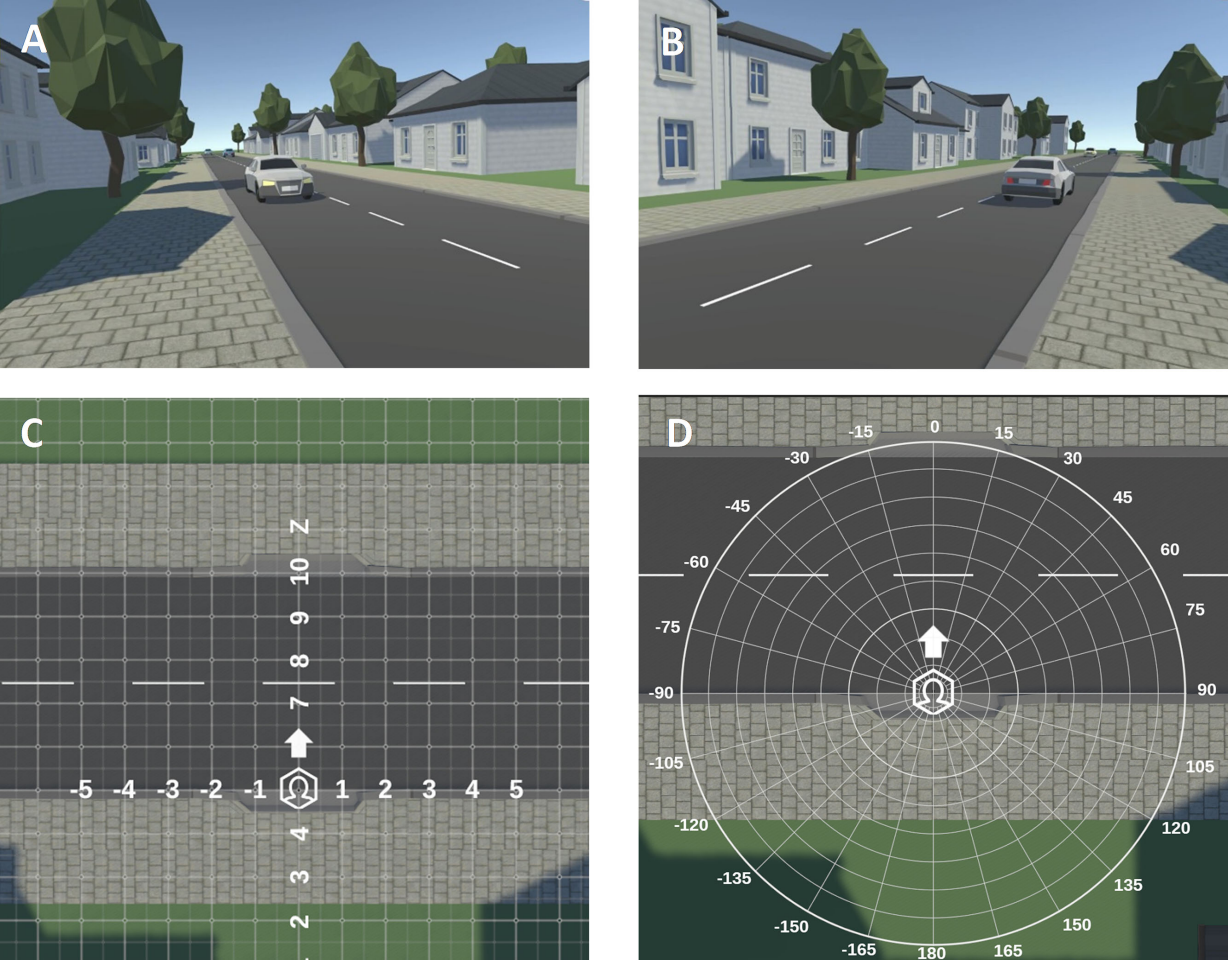
The virtual street crossing environment and its reference system. The virtual street crossing environment from the viewers perspective displaying the right and left side of the scene respectively (**A**–**B**). The virtual street crossing environment consists of a street with two lanes and cars approaching, surrounded by trees and houses in a suburban style. The participants position coordinate system (**C**) in meters along the z-axis (vertical axis) and x-axis (horizontal axis). This coordinate system is used to determine crossing initiation and completion, see data analysis subsection of the method section. The head rotation reference system (**D**) in degrees, which was used to compute the scanning parameters, see data analysis subsection of the method section.

Based on specifications provided by the authors, the custom development in Unity (Unity Technologies, San Francisco, CA, USA) of the VR environment including a hemianopia simulation was outsourced (The Virtual Dutch Men Corporation, Almelo, The Netherlands). The environment simulated a suburban setting encompassing a two-lane street bordered by houses and trees (see Figures 1A and 1B). This suburban setting did not change throughout the experiment and was the same for all participants. The experiment consisted of several phases (Table 2). In all phases cars approached simultaneously from two directions, so the distance between the crossing location and the next approaching car was always similar for the left and right side. This mirrored simulation of the approaching cars aimed to reduce the influence of environmental factors on scanning behavior. More specifically, it minimized interaction effects between environmental factors and the side of the visual field defect; It ensures that the participants perceive the same information on gap size when looking to the left as when looking to the right. The inclusion of the two speed conditions, cars traveling at a speed of either 30 km/h or 50 km/h, allowed for manipulation of speed, as prior research indicated its influence on street crossing decisions in VR (Feldstein & Dyszak, 2020; Schneider et al., 2022). The cars maintained a consistent temporal gap between them or the gap duration increased progressively with each subsequent car, starting with a 3-second interval and increasing by 0.5 seconds per gap (Table 2). Our virtual environment did not include sound, therefore the participants could only rely on their visual input. Figures 1C and 1D illustrate the reference system for measuring head position (in meters) and rotation (in degrees). In this reference system the car lanes are located between 5 to 9.9m on the z-axis (Figure 1C). The simulated distance in the virtual environment was equal to the distance in real-life, preventing any perceptual mismatch between moving in VR and the real world.

**Table 2.** The phases in the VR paradigm. *Notes*: *Asterisk* indicates start time of three seconds between the first two cars, with time increasing 0.5 second after each gap. NB: If a participant did not feel safe to cross in the first trial of practice 1, gap duration would increase with 1 second until the participant felt safe enough to cross. This duration would be maintained through phase practice 2 and 3.

Phase	Gap variant	Gap duration (s)	Car speed (km/h)	Number of crossings
Practice 1	Constant	11	30	2
Practice 2	Constant	8	30	4
Practice 3	Constant	8	50	4
Experimental 1	Increasing	3 + 0.5^*^	30	4
Experimental 2	Increasing	3 + 0.5^*^	50	4

The hemianopia simulation featured a gray area with a hard edge boundary between the visible and blind hemispace, which was superimposed on the display of the head-mounted display. The size and side of the gray area were tailored to each individual with simulated HH based on the visual field defect of their age- and gender matched counterpart with HH. The position of the simulation in the display was dynamically adjusted to the eye-direction provided by the built-in eye-tracker. Based on a previous report including similar hardware, software and sample frequency, we expect a latency of approximately 80ms (Stein et al., 2021).

To assess the possible presence of nausea symptoms during the experiment, the Misery Scale was used (Bos, Mackinnon, & Patterson, 2005). Comprising an 11-point scale, this scale evaluates varying levels of nausea symptoms with corresponding intensities. A score above 6 indicates the presence of severe nausea symptoms, in which case the experiment was terminated. To evaluate participants' simulator sickness after completing the experiment, we used the Simulator Sickness Questionnaire (Kennedy, Lane, Berbaum, & Lilienthal, 1993). This questionnaire includes 16 items with a four-point Likert scale, ranging from no symptoms (0) to severe symptoms (3), to assess symptom severity (maximum item score is 3). Based on previous research, a score of approximately 13 on the SSQ can be expected from virtual reality environments that did not include a mismatch between movement in the virtual and real-life environment (Caserman, Garcia-Agundez, Gámez Zerban, & Göbel, 2021). These authors also suggested that a score of 40 or higher would indicate a bad simulator (Caserman et al., 2021).

### Experimental procedure

The VR experiment took place at different Royal Dutch Visio rehabilitation centers (i.e., Haren, Leeuwarden, Rotterdam, Nijmegen, and Amsterdam). At each center, the experimental room was at least 7 × 4 m, so the participants would be able to walk at least 0.8 m onto the virtual sidewalk on the other side of the street. The head mounted display was affixed to the participant's head, after which it was calibrated. After calibration, a calibration check was conducted that required the participant to sequentially direct their gaze towards seven cones placed at predefined locations in the VR environment. The calibration check was considered successful if the estimated gaze direction did not deviate by more than 2.5° from the designated cone. This was visually confirmed by ensuring that the circle, representing the estimated gaze direction with a radius of 2.5°, overlapped with the cone. If the calibration check was successful, the session commenced with a practice phase. To allow the participant to get used to the VR environment, they first were asked to cross an empty street. This could be repeated as often as they wanted. Once they indicated they felt comfortable with this task, they were asked to cross the empty street two more times. Subsequently, the participant practiced crossing a street with simulated cars in three phases of increasing complexity. In phase 1, performed twice, cars drove at 30 km/h and had a constant 11-sec gap in between them (see Table 2). In phases 2 and 3, each performed four times, cars drove at 30 and 50 km/h, respectively, and had a constant eight-second gap in between them. In case a participant did not consider it safe to cross, gap duration was incrementally extended by 1 sec until the participant crossed the street. The gap duration ultimately set in practice phase 1 was also used in practice phase 2 and 3. Following practice, participants engaged in a total of eight crossings. First, 4 crossings in experimental phase 1 and then 4 crossings in experimental phase 2 (Table 2). The participants in the SH-group completed all phases, except practice 1, with the simulated HH.

In all phases, the participants were instructed to choose the first safe crossing opportunity after the first approaching car had passed. To indicate their decision, they were asked to actually step forward and cross the street at their customary walking pace. Before commencing experimental phases 1 and 2, the participant was informed about the occurrence of a gradual increase in car gap duration. The Misery Scale was administered after the completion of practice phase 2 and experimental phase 1. The experiment was terminated if a participant's score exceeded 6. After experiment completion, the participant filled in the Simulator Sickness Questionnaire.

### Data analysis

The eye- and head-tracking data were analyzed using MATLAB (v2022b; The MathWorks Inc., Natick, MA, USA). The eye-tracking data was provided by the eye tracker in normalized eye-orientation vectors with values between -1 and 1 (Imaoka, Flury, & de Bruin, 2020). Unreliable eye-tracking data were removed based on the data validity measure provided by the eye-tracker. Data gaps of 0.1 seconds or less were filled in using Matlab's shape-preserving cubic spline interpolation (Curve Fitting Toolbox).

**Figure 2.**
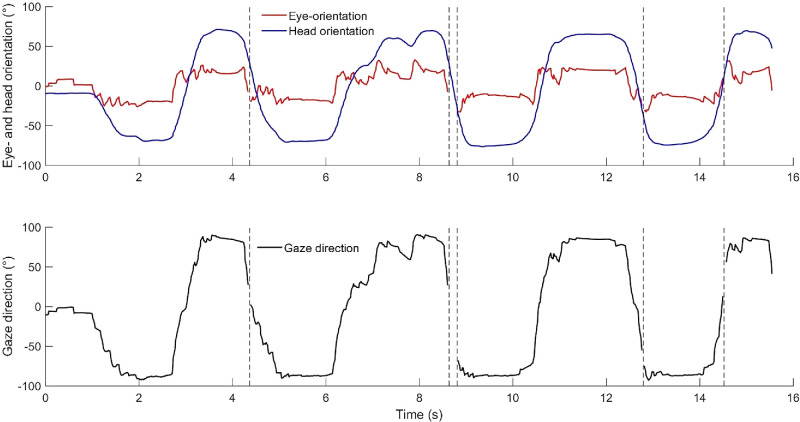
Computation of the horizontal gaze direction. Horizontal eye- and head orientation (upper panel), which are added up to establish the horizontal gaze direction (lower panel) visualized over time. The vertical dotted lines indicate the data gaps.

Upon data gap filling, the normalized eye-orientation vectors were transformed into degrees of the horizontal visual angle within the same reference frame as the head orientation, utilizing Equation 1 (Imaoka et al., 2020). Positive orientation values denote a direction towards the right of the environmental midline, while negative ones indicate a direction towards the left (refer to Figure 1C for the reference system). Lastly, the transformed eye-orientation was added to the horizontal head orientation data to establish horizontal gaze direction (see Figure 2). Because of the missing values in the eye-orientation, there are data gaps in the gaze direction data. We chose not to fill these data gaps using only head orientation data, as we define the gaze direction as the combination of both horizontal eye and head orientation.

(1) $$\begin{array}{c} E_{tran}=\frac{\left(\frac{EN_{tran}}{EN_{long}}\right)\;}{\pi }\;180^{\circ }\; \end{array}$$

where E_tran_ = Horizontal eye-orientation on transverse axis in degrees, EN_tran_ = Normalized eye-orientation on transverse axis, and EN_long_ = Normalized eye-orientation on longitudinal axis.

**Figure 3.**
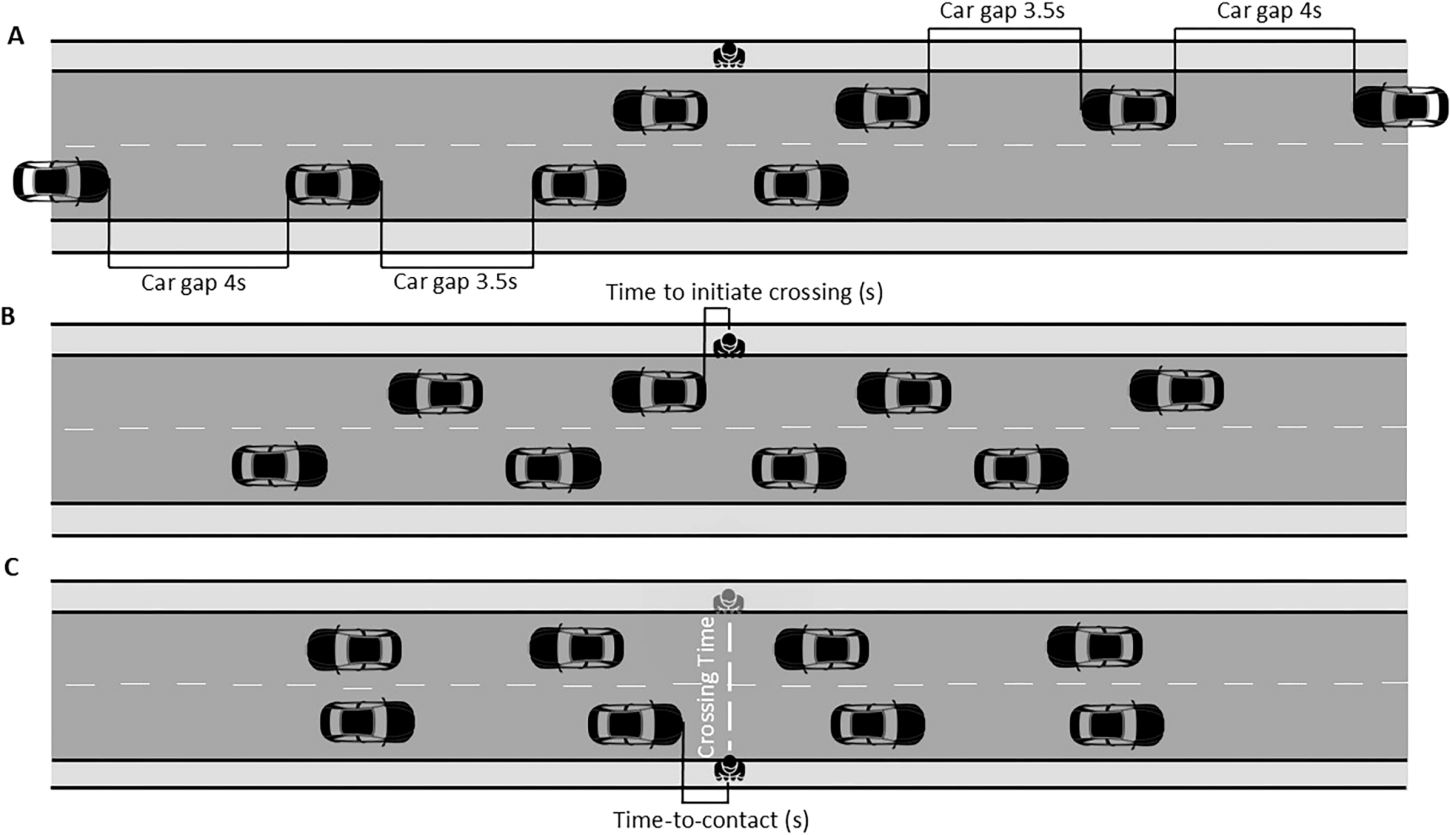
The experimental street crossing paradigm, crossing variables, and crossing outcome*.* Panel **A** illustrates the street crossing paradigm. The car gap duration begins at three seconds and increases in 0.5-second increments with each subsequent gap. Cars approach at either 30 km/h or 50 km/h. The *selected car gap duration* refers to the specific gap duration chosen by participants to cross the streets. Panel **B** shows the *time to initiate crossing,* defined as the interval between the passage of a car at the crossing point and the participant's first step off the curb to start the crossing. Panel **C** presents two additional variables: *crossing time* and *time-to-contact*. Crossing time is the duration between stepping off the initial curb and reaching the opposite curb. *Time-to-contact*, the crossing outcome, is defined as the time between crossing completion and the arrival of the next approaching car at the crossing location.

Crossing outcomes were determined by the variables time-to-contact and the number of collisions. The first crossing outcome parameter, the time-to-contact, was measured as the interval, in seconds, between crossing completion and the next approaching car reaching the crossing location (Figure 3C). Additionally, we recorded the number of collisions encountered by each participant across both car speed conditions as the second crossing outcome variable. The parameter time-to-contact was averaged across the four trials for each car speed condition.

To evaluate crossing behavior, we analyzed the parameters car gap duration of the selected crossing gaps, time to initiate crossing, and crossing time. The median car gap duration (in seconds) was computed across the four trials for each car speed condition (Figure 3A). The time to initiate crossing was defined as the interval, in seconds, between the passage of the car at the crossing location and the participant's initiation of crossing (Figure 3B). Crossing initiation was identified by the participant's step off the sidewalk curb. Depending on the starting position, (i.e., <5 m on the z-axis for one side of the road or beyond 9.9 m on the other side of the road), the stepping off the curb was indicated by a z-axis position exceeding 5 m or falling below 9.9 m (Figure 1C). Crossing time represented the duration from crossing initiation to completion, with crossing completion defined as the participant stepping onto the sidewalk curb on the other side of the street (i.e., participant position along the z-axis was either >9.8 or <5.1 m, as illustrated in Figures 1C and 3C). The parameters time to initiate crossing, crossing time were averaged across the four trials for each car speed condition.

The investigated scanning behavior encompassed head movement frequency, gaze direction toward the blind hemispace, and gaze on task-relevant objects, assessed across two distinct time periods. A previous study showed changes in scanning behavior within 4 secs prior to street crossing (Geruschat, Hassan, Turano, Quigley, & Congdon, 2006). For this reason, we analyzed scanning behavior for two distinct time periods. Time period 1 initiated at the beginning of the trial and concluded four seconds before actual crossing. Time period 2 encompassed the final four seconds before crossing.

To calculate the head movement frequency, we identified horizontal head movements in the head tracking data by detecting velocity peaks of horizontal movements above 50°/s, flanked by preceding and succeeding valleys with velocities of less than 25°/s. The number of head movements was divided by the time frame for each time period separately, computing the head movement frequency in Hz.

The gaze direction towards the blind hemispace during both time periods was assessed following a method similarly to Pundlik et al., (2023). First, we identified the peripheral gaze events defined as intervals in which the gaze direction exceeded >30° (rightward scans) or <−30° (leftwards scans) for at least 135ms (for the reference system see Figure 1D). Subsequently, the gaze peak regions were identified as the highest or lowest 10% of the gaze direction within a given peripheral gaze event. In contrast to the approach of Pundlik et al., (2023), we refrained from calculating the peaks as the gaze direction to mitigate the impact of noise on our results. Instead, we computed the median gaze direction within the gaze peak areas. Next, we selected the calculated median gaze direction of the peripheral gaze events that were directed towards the blind hemispace. For individuals with unimpaired vision, the left or right gaze scans were selected based on the side of the visual field defect of their age- and gender matched counterpart with HH. The selected median gaze direction towards the blind hemispace within was averaged for each crossing trial. The median gaze direction towards the blind hemispace is influenced by the relationship between car direction and the side of the visual field defect. This effect arises from the positioning of car lanes (left lane boundaries: approximately −95° and −65°; right lane boundaries: approximately 60° and 90°; for the reference system, see Figure 1D). Cars coming from the left were driving on the lane closest to the participant, whereas cars coming from the right drove on the far lane. Therefore individuals with left HH would need to direct their gaze further towards their blind hemispace compared to those with right HH when looking at the far end of the lane on their blind side. To account for this disparity, we subtracted the median gaze direction of the unimpaired vision group during the time period prior to the last 4s before crossing from the median gaze direction toward the blind hemispace for each individual. This provided the relative gaze direction, which is not influenced by the relationship between car direction and the visual field defect.

We also determined whether participants allocated their gaze towards task-relevant objects prior to crossing. Gaze-on-cars was defined as the proportion of gaze samples directed towards cars assuming a margin of 4°. For the remainder of the samples we defined whether gaze was directed on the road. Gaze-on-road was defined as the proportion of gaze samples directed towards the car lanes containing approaching cars. The gaze direction boundaries used to determine if gaze was directed toward the left car lane were −95° and −65°, whereas those for the cars lane on the right side were 60° and 90° (for the reference system see Figure 1D). All four scanning parameters (i.e., head movement frequency, gaze direction toward the blind hemispace, and proportion of gaze-on-cars and gaze-on-road) were averaged across the four trials for each car speed condition and time period.

### Statistical analysis

Statistical analysis was conducted using SPSS (v27 IBM SPSS Statistics; IBM, Armonk, NY, USA). To assess the influence of (real or simulated) HH and car speed on crossing outcomes, we conducted a repeated-measures analysis of variance (ANOVA) on the time-to-contact. Participant group (real HH, unimpaired vision, simulated HH) was included as a between-group factor, while car speed (30/50 km/h) was included as a within-group factor with the time-to-contact being the dependent variable. To assess whether car speed differentially affected the time-to-contact across the participant groups, an interaction effect between car speed and group was included in the analysis. Post-hoc tests were conducted when a significant effect was detected for the main-effect of the factor group or the interaction-effect between the factors group and speed. To assess a difference in the number of collisions due to hemianopia, a chi-square test was included comparing the number of participants who did and did not encounter collisions across the three groups.

To assess the influence of (real or simulated) HH and car speed on crossing behavior, a repeated-measures ANOVA was employed with the crossing parameters (i.e. median selected car gap duration, time to initiate crossing, and crossing time) as dependent variables. The same factors and interaction-effects as previous analysis were included. Post-hoc tests were conducted when a significant effect was detected for the main-effect of the factor group or the interaction-effect between the factors group and speed.

For exploring group differences in scanning behavior before crossing a street, repeated measures ANOVA were performed for the four scanning parameters, head movement frequency, relative gaze direction toward blind hemispace, proportion of gaze-on-cars, and proportion of gaze-on-road. These analyses included next to the factors of the first analyses (i.e., between-group factor participant group, and within-group factor car speed), the two time periods (1 or 2) as a within-group factor, the two-way interaction-effect between group and time period, and the three-way interaction effect between group, speed, and time period. The factor time period was added to examine whether scanning behavior changed in the final four seconds before crossings, and whether these changes depended on simulated car speed and differed between participant groups.

When a parameter did not adhere to a normal distribution, we used nonparametric analyses. However, traditional nonparametric methods lack the capability to incorporate interaction effects. Therefore, to replace the original repeated measures ANOVA, we transformed the data into ranking scores using the Aligned Transformation Tool (Wobbrock, Findlater, Gergle, & Higgins, 2011). Subsequently, these ranking scores were subjected to a repeated measures ANOVA, enabling the inclusion of the interaction effects. For analysis assessing group differences without interaction effects (i.e., independent *t*-test), we substituted them with the Mann-Whitney U test.

A significance level of 0.05 was adhered to for all analyses. When performing repeated measures ANOVAs, we computed the partial eta squared effect-size with 0.01 as small, 0.06 as medium and 0.14 as large effect-size. We defined trends as statistical outcomes with a large effect-size, but no significant *p* value.

## Results

### Data quality

One participant with real hemianopia only completed the trials with cars traveling at 30 km/h because of a walking impairment. Because none of the participants scored above 7 on the Misery Scale, none of them had to be excluded due to nausea. The scores on the Simulator Sickness Questionnaire after experiment completion were on average 17 (*SD* = 15) for the real HH group, 17 (*SD* = 15) for the unimpaired vision group, and 19 (*SD* = 18) for the simulated HH group. On average, we recorded 203 seconds (3.4 minutes) of data per participant for the four trials in which cars traveled 30 km/h, and 112 seconds (1.8 minutes) for the total of four trials in which cars traveled 50 km/h. The average data loss per participant was 9.3%, with a standard deviation of 5.1%. This data loss consisted of eye-tracking data samples evaluated as unreliable indicated by the data validity measure provided by the eye-tracker. The accuracy of the eye-tracker data, as evaluated by a calibration check, was on average 0.99° with a range of 0.39°–2.52°.

### Crossing outcomes

**Figure 4.**
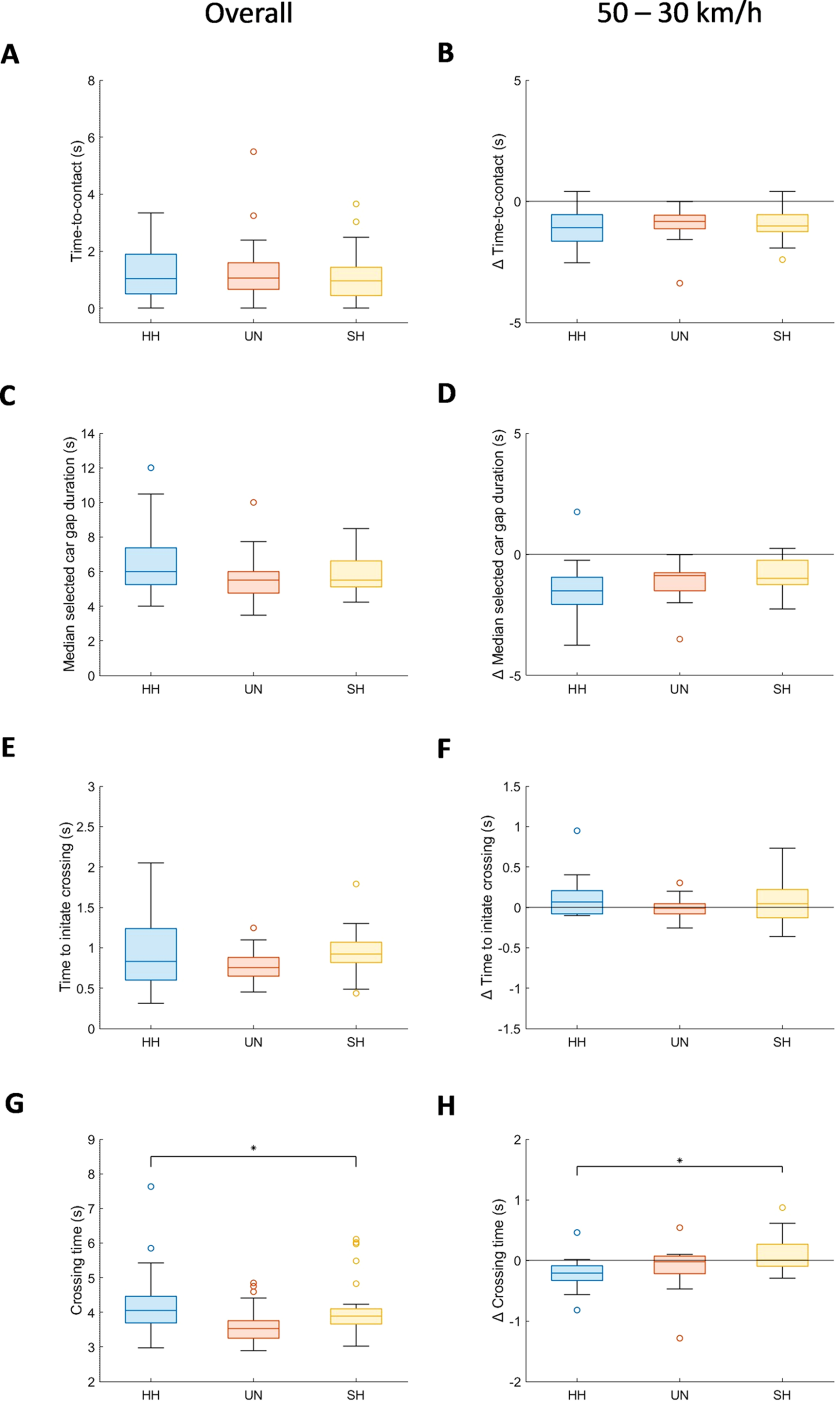
Crossing outcomes and behavior across participants groups and speed conditions. The left panel shows the overall group comparison, while the right panel depicts the difference scores between speed conditions. The crossing outcome parameter, time-to-contact, is depicted in **A**–**B**. The presented crossing behavior parameters are (**C**–**D**) car gap duration, (**E**–**F**) time to initiate crossing, and (**G**–**H**) crossing time. In the right panel, a score above zero would indicate that the parameter, for example time-to-contact, increases when cars travel 50 km/h, whereas a score below zero would indicate a decrease in the parameter when cars travel 50 km/h.

**Figure 5.**
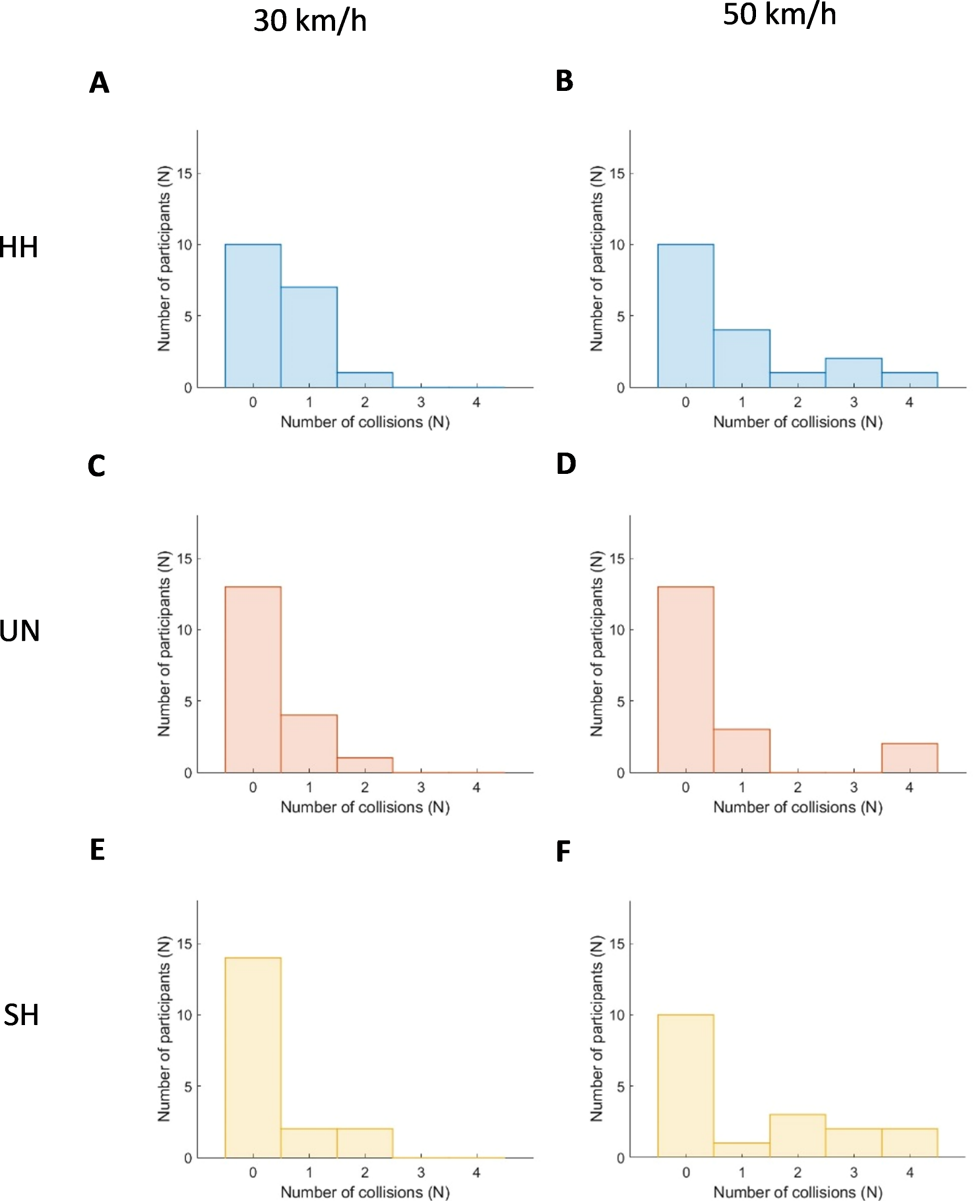
The number of collisions encountered across the participant groups and car speed conditions. The histograms show the number of participants who encountered zero to a maximum of four collisions, separated per group and car speed (left panel: 30 km/h; right panel: 50 km/h). The participant groups included (**A**–**B**) individuals with real homonymous hemianopia (HH), (**C**–**D**) those with unimpaired vision (UN) and (**E**–**F**) those with simulated homonymous hemianopia (SH).

Figures 4A and 4B depicts the overall group comparison and difference scores between speed conditions for the crossing outcome parameters; time-to-contact. Figure 5 shows the number of participants involved in one or more collisions. Additionally, Supplementary Figure A3D presents the crossing outcomes per group and condition.

The time-to-contact did not differ between groups (i.e., no significant group-effect, and a small effect size, Table 3; Figure 4A). Additionally, there was no difference found in the number of participants experiencing collisions across groups (Table 4; Figure 5). Car speed influenced crossing outcome. Figure 4B demonstrate that participants show a shorter time-to-contact when cars traveled 50 km/h compared to 30 km/h (i.e., significant main-effects of speed and large effect sizes). The effect of car speed on the time-to-contact was consistent across groups, demonstrated by the absence of a group by speed interaction-effect.

**Table 3.** Comparison of crossing behavior and outcome between groups and car speed. *Notes*: Significant results are presented in bold. *Asterisk* indicates large effect size.

	Time-to- contact (s)	Car gap duration (s)	Crossing time (s)	Time to initiate crossing (s)
Main-effect				
*F*(2,50)	0.333	2.31	**3.51**	1.90
*P*	0.718	0.110	**0.037**	0.160
η²	0.013	0.085	**0.123**	0.071
Main-effect speed				
*F*(1,50)	**97.1**	**85.7**	2.44	3.15
*p*	**<0.001**	**<0.001**	0.125	0.082
η²	0.660^*^	0.631^*^	0.046	0.059
Interaction-effect speed^*^				
*F*(1,50)	0.313	1.34	**4.78**	0.965
*p*	0.733	0.270	**0.013**	0.388
η²	0.012	0.051	0.160^*^	0.037

**Table 4.** Statistical comparison of the number participants engaged in at least one virtual collision (yes) versus no collisions (no) between groups. *Notes*: HH = homonymous hemianopia; UN = unimpaired vision; SH = simulated homonymous hemianopia.

	N	χ^2^ (2,54)	*p*
30 km/h		2.23	0.328
HH			
Yes	8		
No	10		
UN			
Yes	5		
No	13		
SH			
Yes	4		
No	14		
50 km/h		1.62	0.444
HH			
Yes	8		
No	9		
UN			
Yes	5		
No	13		
SH			
Yes	8		
No	10		

### Crossing behavior

Figures 4C–H depicts the overall group comparison and difference scores between speed conditions for the crossing behavior parameters (i.e. median selected car gap duration, time to initiate crossing and crossing time). Supplementary Figure A1 illustrates the total number of crossings as a function of car gap duration. We also included the total number of crossings as a function of the selection of car gap distances in the Appendix (Supplementary Figure A2). Additionally, Figures 3A to 3C present the crossing behavior parameters per group and condition.

Regarding crossing time, differences were found between three groups, which were not consistent across the two car speeds (Figures 4G and 4H). This is indicated by a group main-effect and a group*speed interaction-effect with a medium and high effect size respectively (Table 3). Post-hoc tests revealed that participants with HH took a longer time to cross compared to participants with unimpaired vision when the cars traveled at 30 km/h (*p* = 0.039). This effect was less visible when cars traveled 50 km/h (*p* = 0.111). Individuals with simulated HH took a longer time to cross than those with unimpaired vision when cars traveled 50 km/h (*p* = 0.039), but not when cars traveled 30 km/h (*p* = 0.414). There was no difference in crossing time between individuals with real and simulated HH at both car speeds (*p* > 0.83). No group differences were observed for the other crossing behavior parameters (i.e. no significant group-effect, and small effect size, Table 3).

Car speed also influenced crossing behavior. Figure 4D demonstrates that participants select shorter gap durations when cars traveled 50 km/h compared to 30 km/h (i.e., significant main-effect of speed and large effect size). The effect of car speed on car gap duration was consistent across groups, demonstrated by the absence of a group by speed interaction-effect.

### Scanning behavior and the influence of car speed

**Figure 6.**
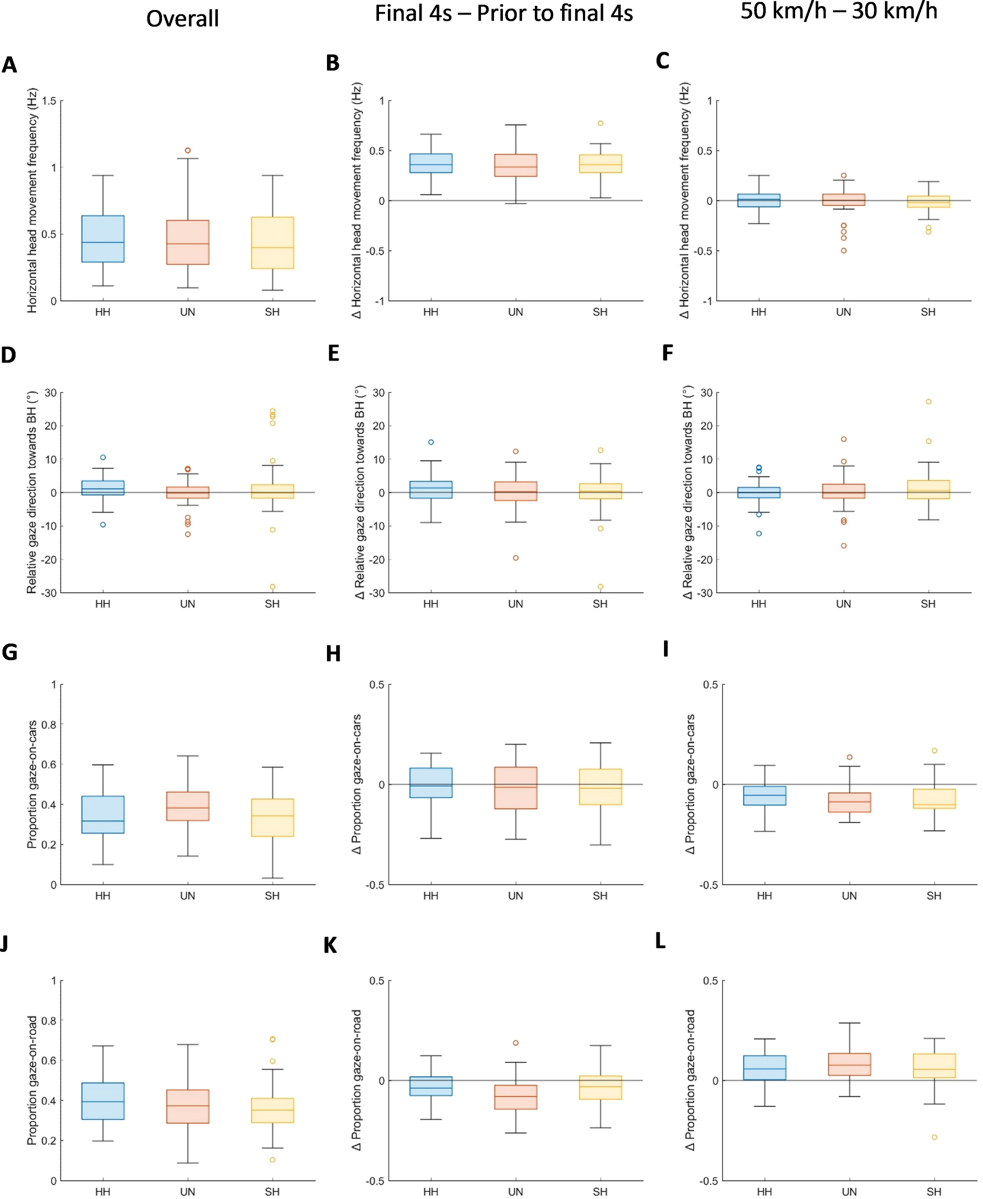
Scanning behavior across participants groups and speed conditions. The left panel shows the overall group comparison, the middle panel illustrates the difference scores between scanning behavior employed at the final four seconds before crossing and before these final four seconds, and the right panel depicts the difference scores between speed conditions. The presented scanning behavior parameters are (**A**–**C**) head movement frequency, (**D**–**F**) relative gaze direction toward blind hemispace (BH), (**G**–**I**) proportion gaze-on-cars, and (**J**–**L**) proportion gaze-on-road. In the middle panel, a score above zero would indicate that the parameter, for example horizontal head movement frequency, increases in the final four seconds before crossing, whereas a score below zero would indicate a decrease in the parameter in the final four seconds before crossing. In the right panel, a score above zero would indicate that the parameter, for example horizontal head movement frequency, increases when cars travel 50 km/h, whereas a score below zero would indicate a decrease in the parameter when cars travel 50 km/h.

Figure 6 illustrates the overall group comparison, difference scores between time periods and difference scores between speed conditions for the scanning behavior parameters (i.e. head movement frequency, relative gaze direction towards blind hemispace, gaze-on-cars and gaze-on-road). Supplementary Figure A4 illustrates the scanning behavior (i.e., head movement frequency, relative gaze direction towards blind hemispace, gaze-on-cars and gaze-on-road) across the three groups, the two car speeds, and both time periods. Supplementary Figure A5 depicts the rankings score used for the statistical analysis of the relative gaze direction toward the blind hemispace.

Overall, three groups do not differ in scanning behavior, as indicated by the absence of a main-group effect or high effect sizes (Table 5). In the final four seconds before crossing, the participants increased their head movement frequency, reduced their proportion of gaze-on-road, and showed a slight increase in their relative gaze direction toward the blind hemispace. Yet, these changes were consistent across groups. This is demonstrated by a main-effect of a time period with a high or medium effect size and the absence of a time period by group interaction effect (Table 5). The proportion of gaze-on-cars remained similar across the two time periods, indicated by an absence of a time period main-effect and time period by group interaction-effect (Table 5).

**Table 5.** Statistical comparison of scanning behavior between groups, time period and car speed. *Notes*: Significant results are presented in bold. *Asterisk* indicates Data transformed to ranks with the aligned transformation tool (Wobbrock et al., 2011). *Dagger* indicates high-effect sizes.

	Head movement frequency (Hz)	Relative gaze direction towards blind hemispace (°)^1^	Gaze-on-cars (%)	Gaze-on-road (%)
Main-effect				
*F*(2,51)	0.432	1.06	1.79	0.819
*P*	0.652	0.355	0.177	0.447
η²	0.017	0.041	0.067	0.032
Main-effect time period				
*F*(1,51)	**354**	**4.64**	1.23	**28.1**
*P*	**<0.001**	**0.036**	0.273	**<0.001**
η²	0.878^*^	**0.085**	0.024	0.360^*^
Interaction-effect time period^†^				
*F*(2,51)	0.140	0.757	0.086	2.08
*P*	0.870	0.474	0.918	0.136
η²	0.878	0.029	0.003	0.077
Main-effect speed				
*F*(1,51)	0.848	0.483	**74.1**	**49.9**
*P*	0.362	0.490	**<0.001**	**<0.001**
η²	0.017	0.010	0.597^*^	0.500^*^
Interaction-effect speed^†^				
*F*(1,52)	0.368	1.09	0.308	0.984
*P*	0.694	0.344	0.736	0.381
η²	0.015	0.042	0.012	0.038
Interaction-effect speed time period^†^				
*F*(1,51)	0.609	1.21	0.183	0.439
*P*	0.548	0.306	0.833	0.647
η²	0.024	0.046	0.007	0.017

When cars traveled 50 km/h, all participant groups similarly decreased their proportion of gaze-on-cars, while increasing their proportion of gaze-on-road compared to when cars traveled 30 km/h. This is indicated by the speed main-effect with a high effect size and the absence of speed by group interaction-effect. The different car speed conditions did not alter any other scanning parameters, supported by the absence of a speed main-effect and a speed by group interaction-effect (Table 5).

## Discussion

In this study, we explored street crossing outcomes, crossing behavior, and scanning behavior of individuals with either real HH, simulated HH, or unimpaired vision by presenting a virtual crossing environment by means of a head-mounted display. Our main finding is that although both real and simulated HH increases crossing time compared to unimpaired vision, the crossing outcomes in terms of time-to-contact and the number of collisions remain similar. We did not find evidence that the real or simulated HH affected scanning behavior within our virtual environment. Participants altered their scanning behavior in the final four seconds before crossing, yet these adjustments were consistent across all groups. Additionally, the velocity of the simulated cars influenced crossing outcomes, crossing behavior and scanning behavior, but these differences were also consistent across groups.

We observed prolonged crossing times in individuals with real and simulated HH compared to those with unimpaired vision depending on the car speed. Moreover, the crossing times of individuals with simulated HH closely resembled those of individuals with real HH. This similarity in prolonged crossing times implies that the observed effect may primarily stem from the visual field defect rather than from comorbidities of the acquired brain injury. Additionally, the presence of prolonged crossing times in individuals experiencing simulated HH for a short period of time, suggest that such behavior can be present in the early stages after HH onset. Previous studies have suggested that simulations of HH can induce alteration in scanning behavior that arise from vision loss independent of the comorbidities of the acquired brain injury and may be present at the acute stage of HH (Biebl et al., 2022; Tant et al., 2002b). Prior studies have demonstrated that a simulated decrease in contrast sensitivity or blurry vision, reduces walking speed during obstacle avoidance (Vivekananda-Schmidt, Anderson, Reinhardt-Rutland, & Shields, 2004; Zult, Allsop, Timmis, & Pardhan, 2019). This reduction in speed has been proposed to facilitate the acquisition of task-relevant information, enhancing visuomotor planning (Zult et al., 2019). Although HH is a different type of visual impairment, reducing walking pace may afford sufficient time to gather the essential visual information necessary for safe navigation.

Although a reduced walking pace may facilitate visuomotor planning, it also increases the risk of collision. However, we found that the time-to-contact and the number of collisions remained similar across groups. The absence of consistent group-level differences in gap selection or initiation time between groups suggests that individuals use varying crossing strategies to mitigate the effect of their visual impairment resulting in a reduced walking pace. Some may select longer car gap durations, whereas others may initiate their crossing more promptly. Overall, these results highlight that both individuals with real and simulated HH demonstrate adaptive compensatory crossing behavior, essential for successful virtual street crossing.

The presence of HH, whether simulated or real, did not affect scanning behavior. Prior research comparing scanning behavior in individuals with HH to those with unimpaired vision during mobility tasks has yielded mixed results (Postuma et al., 2024; Bowers et al., 2014; Iorizzo et al., 2011; Xu, Baliutaviciute, Swan, & Bowers, 2022). Individuals with HH exhibited a higher proportion of head movements toward their blind hemispace compared to those with unimpaired vision when crossing intersections while driving (Bowers et al., 2014). Yet, the total number of head movements did not differ. In addition, the number of fixations on environmental objects showed no significant differences between individuals with HH and those with unimpaired vision when they navigated a route while detecting moving objects in a virtual environment (Iorizzo et al., 2011). Another simulated driving study found that the extent of differences in fixation durations on intersection cross traffic between the blind and visible hemispace or between individuals with HH and those with unimpaired vision depended on the specifics of the scenario (Xu et al., 2022). All these studies addressed mobility-related tasks, such as detecting moving objects while walking (Iorizzo et al., 2011) or crossing intersections while driving (Bowers et al., 2014). Because of the task-specificity of scanning behavior in individuals with HH (Postuma et al., 2024), a direct comparison to our findings should only be made with caution.

Irrespective of these prior results, similar scanning among the groups could also be specific to street crossing or attributed to the constraints of the current virtual crossing environment. From infancy, each individual has typically been taught to scan both the left and right side of a street before crossing by making head movements. Such prototypical behavior may already suffice, also in case of HH. The task constraints may also have caused the absence of marked differences in scanning behavior, as the scanning patterns of individuals with HH are known to be sensitive to such constraints (Iorizzo et al., 2011; Postuma et al., 2024; Xu et al., 2022). In the present experiment, these constraints include a relatively low variability and high predictability. Although these conditions might have the advantage of capturing fundamental compensatory behavior, they may have reduced the need for more active compensatory scanning to successfully cross the virtual street. In fact, during real-life crossing, characterized by higher variability and unpredictability, individuals with HH have been observed to more frequently perform gaze scans towards their blind hemispace and direct their gaze slightly further into their blind hemispace compared to their visible hemispace (Pundlik et al., 2023), a possible sign of compensatory behaviour.

### The future of using virtual reality to study street crossing and the implications for rehabilitation

By using virtual reality, we were able to study street crossing outcomes, crossing behavior and scanning behavior in a safe, standardized and controllable environment, which is not feasible in real-life. As such, virtual reality in its current form may play a role in rehabilitation (e.g., to accustom those with HH to a task like this). Obviously, it is important to acknowledge that one should be cautious when translating our virtual outcomes to real-life situations. The inability to accurately perceive car speed, impaired depth perception, the reduced field of view, and the relatively low variability and high predictability of the street crossing scenarios, render our virtual street crossing environment distinct from real-life. Therefore improvements to the virtual street crossing paradigm are imperative to enhance its ecological validity and translatability.

First, our findings demonstrate that virtual car speed influences crossing behavior, crossing outcomes, and the associations between scanning behavior and crossing outcomes. This effect can be partly attributed to a decrease in the ability to perceive car speed in a virtual setting (Feldstein & Dyszak, 2020; Schneider et al., 2022). Improving virtual car speed perception may be achieved by increasing the display resolution to smooth the representation of virtual cars (Feldstein, 2019), extending the arrival duration of road-users (Andersen & Enriquez, 2006), including sound (Oberfeld, Wessels, & Büttner, 2022), and modifying the visual information available, such as the rate of optical expansion of approaching cars (Rolin, Fooken, Spering, & Pai, 2019) or increasing the contrast between the road-users and the background (Feldstein & Peli, 2020).

In addition to speed perception, depth perception in VR is also impaired, which may have influenced crossing behavior. This impairment could stem from factors such as the low resolution of display lenses, a reduced field of view, the weight of the headset, and the vergence-accommodation conflict inherent in VR (Kelly, 2023; Rzepka et al., 2023). Improving depth perception in VR street crossing may be achieved by incorporating objects of familiar sizes near the road to facilitate depth cues or through advancements in display technology, such as multifocal lenses (Rzepka et al., 2023; Scarfe & Glennerster, 2019).

The limited field of view should be increased, since it may prompt individuals to rely more on head movements rather than eye movements to scan their surroundings completely (Berton, Olivier, Bruneau, Hoyet, & Pettre, 2019; Berton, Hoyet, Olivier, Bruneau, Le Meur, & Pettre, 2020; Drewes, Feder, & Einhäuser, 2021; Pfeil, Taranta, Kulshreshth, Wisniewski, & LaViola, 2018; Postuma, Cornelissen, Pahlevan, Heutink, & de Haan, 2025). This constrains the number of scanning parameters that could be meaningfully reported.

Last, the limited variability and relatively high predictability of our virtual environment may have reduced the need for active compensatory scanning behavior. Low variability and high predictability are general features of current virtual street crossing environments (Morgan, Stager, Schwebel, & Shen, 2023; Schneider & Li, 2020) and can be resolved by adding various road users, increasing the variability in gap durations between road-users, including objects obstructing the participant's view, and programming road-users who behave in a more unpredictable manner.

Given these limitations, our results should be interpreted in relation to the participant groups within our virtual street crossing paradigm instead of directly reflecting real-life behavior. It is important to note that we aimed to create a street-crossing simulation that captures the essence of street crossing while ensuring sufficient task demands instead of a simulation that closely mimics real-life street crossing. Additionally, we did not focus on measuring the safety of street crossing. This study should be viewed as a stepping stone for future research that may bridge the gap between virtual and real-life street crossing. Such future research could establish whether the absence of compensatory scanning behavior in our experiment is specific to street crossing or due to the limitations of the current virtual street crossing paradigm.

Although the current virtual street crossing paradigm has limitations, it may still hold potential for studying and training street crossing behavior in individuals with HH in the future. Rather than addressing street crossing as a unified skill, a virtual environment could be used to target and train specific components essential for safe street crossing. For instance, a VR paradigm could be used to assess the impact of crowd density and unpredictability in street crossing situations on scanning behavior and its relationship to crossing outcomes. Additionally, individuals with recently acquired HH could benefit from using a virtual street crossing environment during rehabilitation to build confidence in their visuomotor abilities. It seems thereby essential to inform individuals with HH about a potential reduction in their walking pace, and the need to align their street crossing decisions to their visuomotor capabilities. However, before such studies or training methods can be effectively implemented, the aforementioned limitations must be addressed.

### Limitations

As previously mentioned, the relatively low variability and high predictability, the inability to accurately perceive car speeds and the limited field of view are limitations of our virtual environment. We expect that the latter had a limited effect on the scanning parameters reported, because we used the gaze direction data to calculate most scanning variables instead of eye or head orientation. Furthermore, head movement frequency was likely unaffected, because shifting gaze between approaching cars from the left and right side would require performing a head movement even with a natural human field of view. Nevertheless, the limited field of view constrained us in the scanning parameters that could reliably be reported. As a result, we excluded parameters such as head movement amplitude, saccadic amplitude or saccadic frequency. In this study, unreliable eye-tracking data were removed based on the data validity measure provided by the eye tracker. To our knowledge, no studies have yet examined the reliability of the Tobii validity metric. This lack of knowledge poses a limitation to our study. Nevertheless, based on visual inspection, we observed that most of the data loss flagged by the validity metric is likely due to the eye tracker's difficulty in detecting the pupil during rapid eye movements. Based on a previous report involving similar hardware, software, and sampling frequency, we anticipate a latency of approximately 80 ms (Stein et al., 2021). This is relatively high compared to the other head-mounted displays examined (Stein et al., 2021), which showed latencies between 45 ms and 57 ms. Also, compared to gaze contingent studies that e.g. used an Eyelink eye tracker with a display (e.g., Cornelissen, Bruin, & Kooijman, 2005), the delay is relatively long. Theoretically, a relatively long latency could have influenced the results in the simulated HH group. However, we did not observe significant differences in scanning patterns between individuals with real or simulated HH suggesting such an influence was at most very small. Moreover, engaging in an extensive immersive environment, rather than focusing all of one's attention on a relatively small display, may influence whether participants notice screen updates given various delays. With further improvements in VR-based eye tracking hardware, latencies will likely further be reduced. We did not include any variation in the order of speed conditions. Therefore the exposure time to the virtual environment could have influenced the difference in crossing and scanning behavior between the speed conditions. We do not expect that simulator sickness has influenced our outcomes, as the simulator sickness scores were similar across the groups. These scores resemble the scores previously observed in virtual reality environments that did not include a mismatch between movement in the virtual and real-life environment (Caserman et al., 2021). Last, in our experiment, we also observed some apparent gaze changes that can be attributed to environmental alterations rather than shifts in behavior. The car gap durations were kept constant between the two car speed conditions, resulting in longer car gap distances when cars traveled faster and thus a lower car density. Consequently, we found an increased proportion of gaze allocated toward the road and a decreased proportion of gaze allocated toward the cars when cars traveled 50 km/h.

## Conclusions

In summary, in a virtual reality environment, both individuals with real and simulated HH used compensatory crossing strategies to align their crossing behavior to their visuomotor capabilities. Although the visual field defect appeared to reduce their walking pace, they adopted varying compensatory crossing behavior strategies, resulting in similar crossing outcomes to those of individuals with unimpaired vision. HH did not alter scanning behavior before crossing a virtual street. Future research should establish whether this absence of compensatory scanning behavior is specific to street crossing or is specific to the use of a virtual environment. Additionally, virtual crossing paradigms should be enhanced by improving virtual car speed perception, depth perception, extending the field of view, and increasing variability and unpredictability. Despite its current shortcomings, the virtual paradigm still holds promise for street crossing research, offering a safe, standardized and controllable environment to study behavior. Consequently, our insights may serve as a stepping stone toward translational use of VR to identify compensatory crossing and scanning strategies for successful street crossing in individuals with HH.

## Supplementary Material

Supplement 1
