# Supplementary material for: Virtual street crossing and scanning behavior in people with hemianopia: A step toward successful crossings
Source: J Vis. 2025 Sep 2;25(11):1. doi: 10.1167/jov.25.11.1 (PMC12410284; doi:10.1167/jov.25.11.1)
Supplement: Supplement 1 [file jovi-25-11-1_s001.pdf]

Appendix

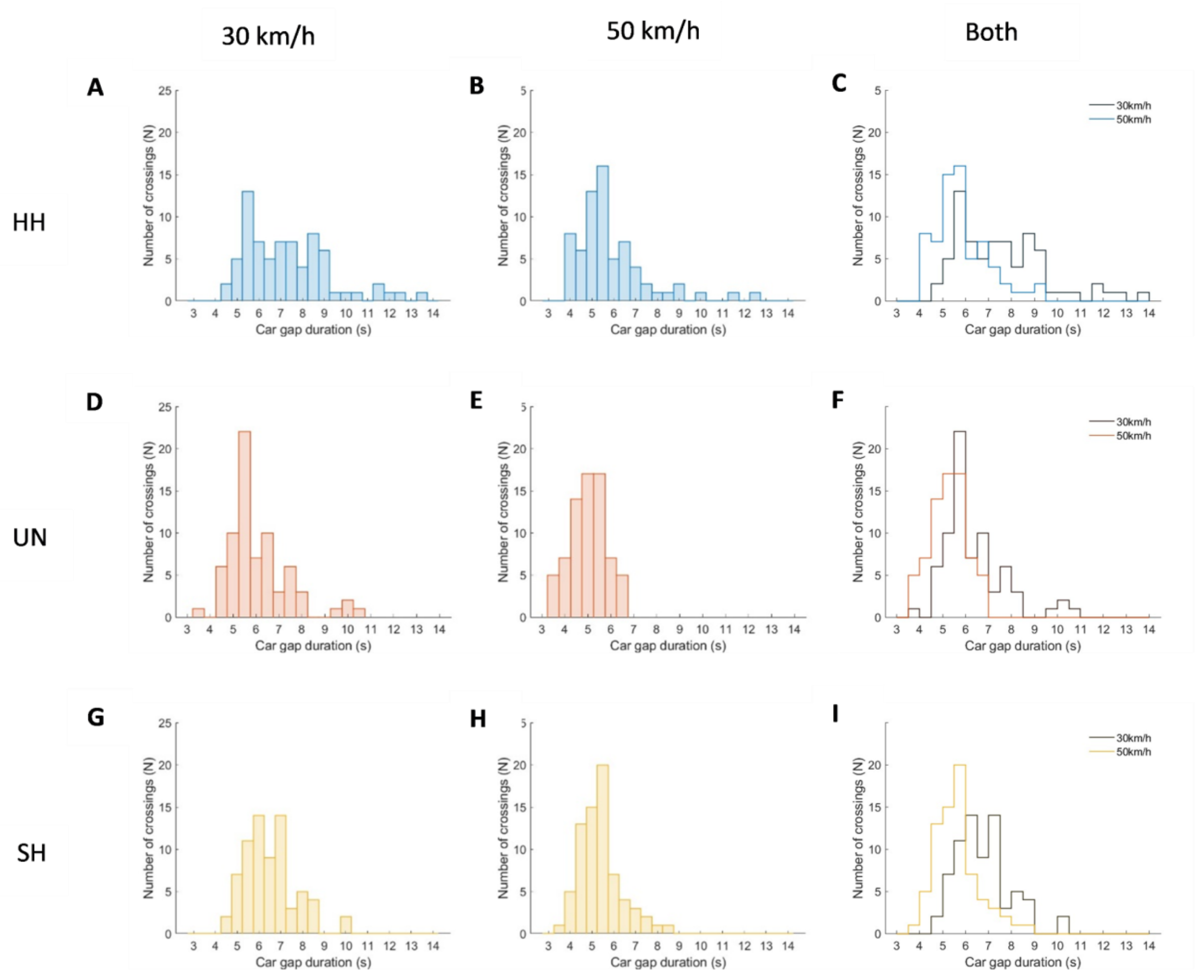

Figure Appendix 1. **The selected car gap duration across participant groups and car speed conditions.** Histograms showing the total number of all crossings made as a function of car gap duration, split per group (rows) and car speed (columns).

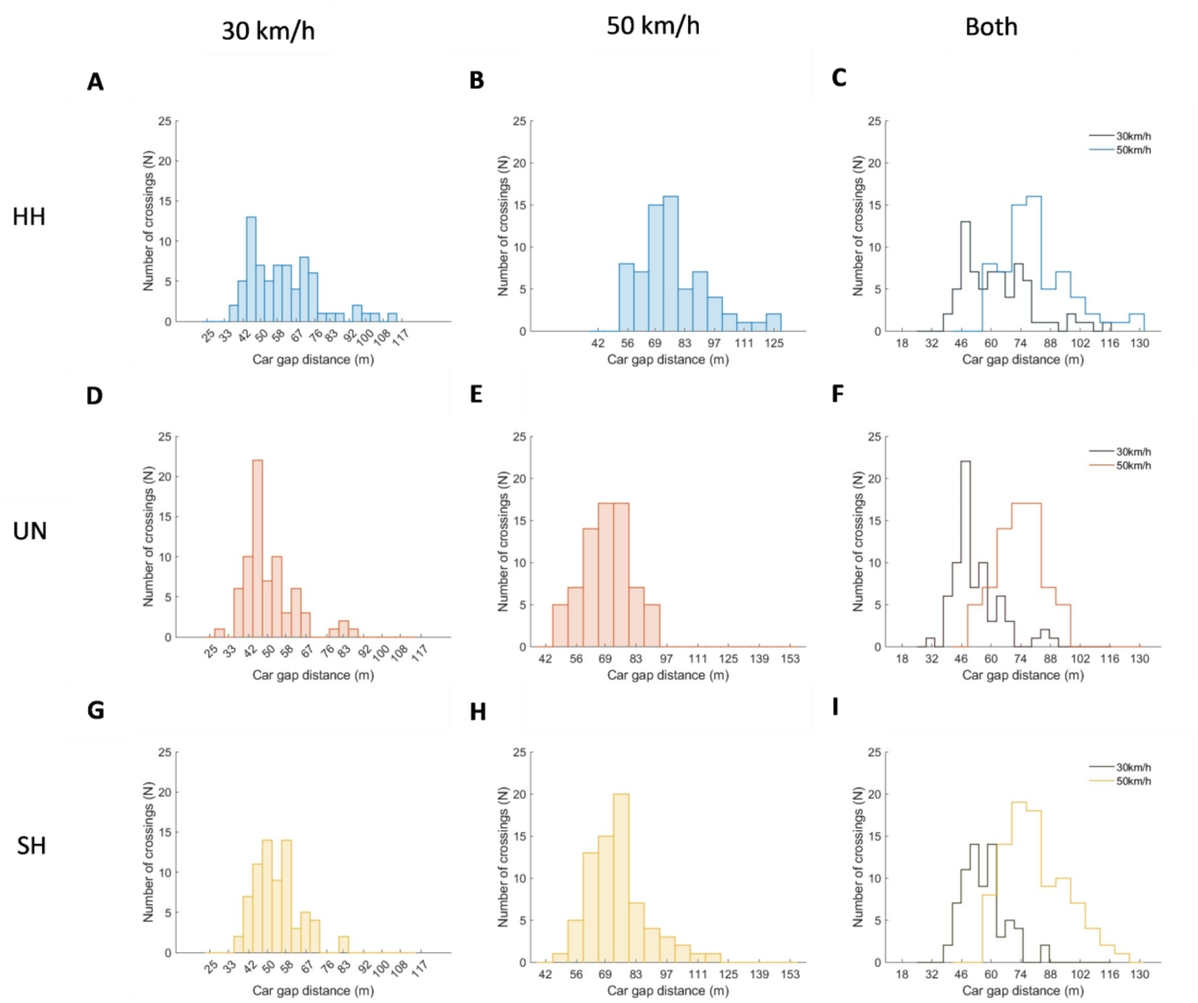

Figure Appendix 2. **The selected car gap distance across participant groups and car speed conditions.** Histograms showing the total number of all crossings made as a function of car gap distance, split per group (rows) and car speed (columns).

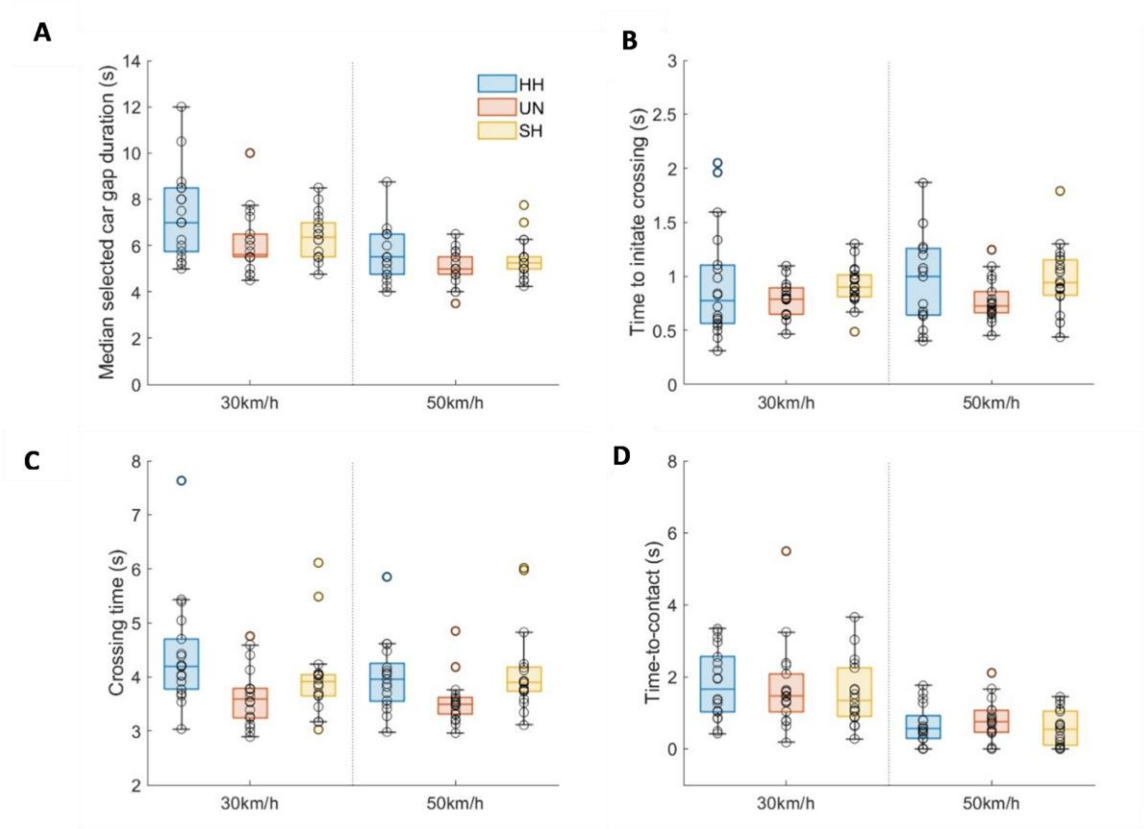

Figure appendix 3. ***Crossing behavior parameters and the crossing outcome parameter.*** Crossing behavior (A-C) and outcomes (D) per participant group and car speeds. The presented variables are (A) car gap duration, (B) time to initiate crossing, (C) crossing time, and (D) time-to-contact. For this and all following figures, data for participants with real homonymous hemianopia (HH) are shown in blue, for those with unimpaired vision (UN) in red, and for those in whom we simulated hemianopia (SH) in yellow.

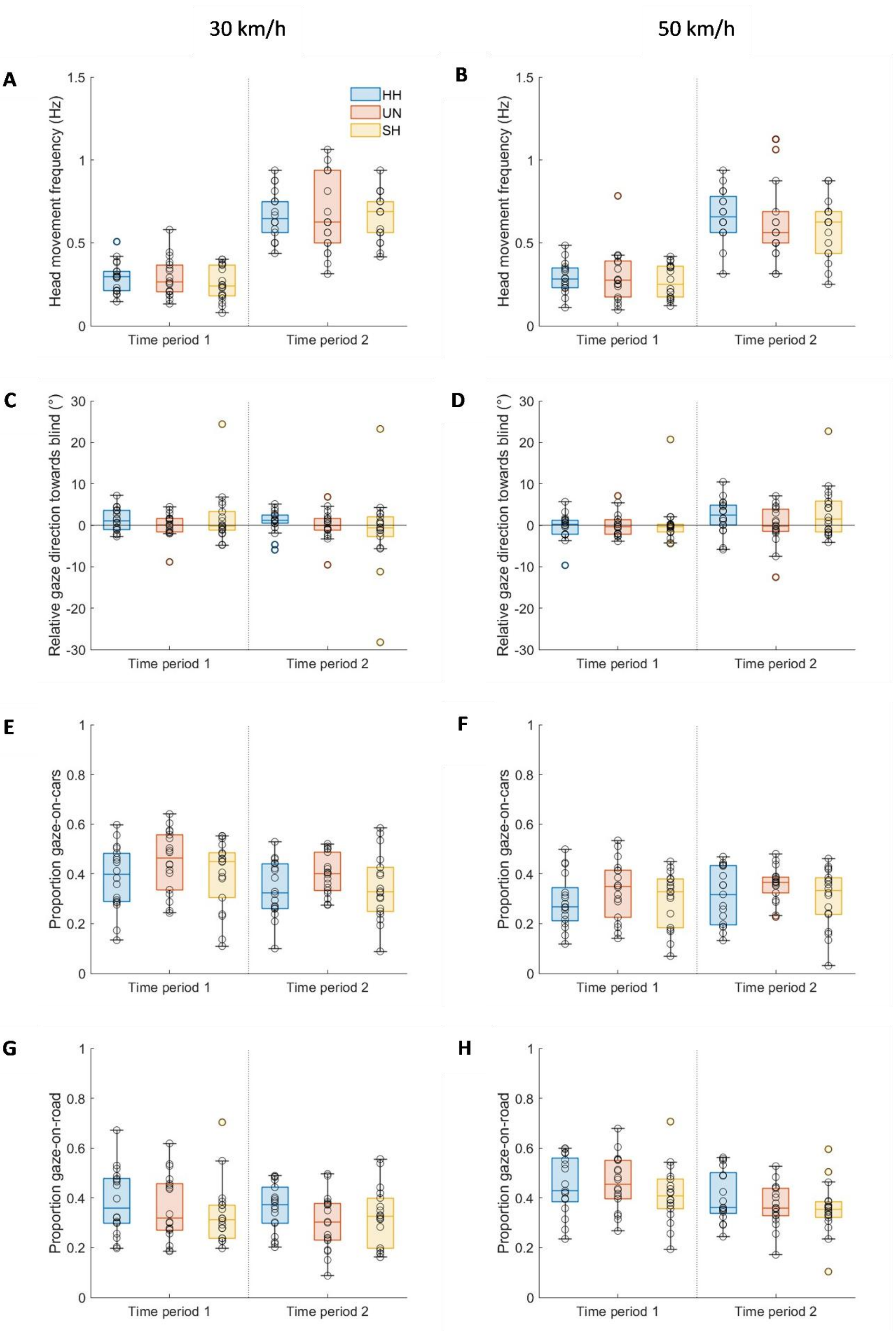

30 km/h
50 km/h
A
B
C
D
E
F
G
H
HH
UN
SH
Head movement frequency (Hz)
Relative gaze direction towards blind (°)
Proportion gaze-on-cars
Proportion gaze-on-road
Time period 1
Time period 2

Figure Appendix 4. **Scanning behavior parameters across groups, time period and car speeds.** Scanning behavior per group, time period and car speed for (A&B) head movement frequency (Hz) (C&D) Relative gaze direction towards blind hemispace (°), (E&F) proportion of gaze-on-cars, and (G&H) proportion of gaze-on-road. Scanning behavior is displayed for time period 1 (i.e. scanning behavior until the last four seconds before crossing) and time period 2 (i.e. scanning behavior during the last four seconds before crossing). Additionally, participant's scanning behavior during the condition where cars traveled 30 km/h is illustrated in the left panel, and the condition where cars traveled 50 km/h is illustrated in the right panel. The participant groups included individuals with real homonymous hemianopia (HH), those with unimpaired vision (UN) and those with simulated homonymous hemianopia (SH).

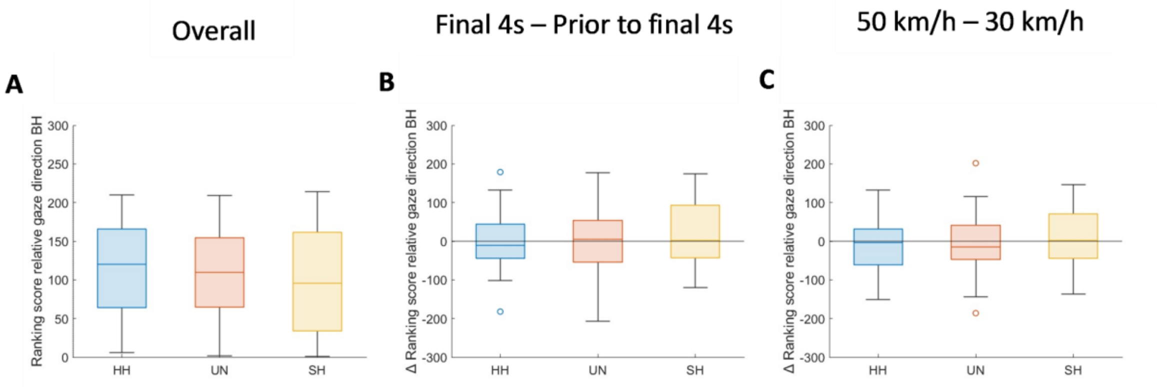

Figure appendix 5. **Ranking scores of the relative gaze direction towards the blind hemispace.** The left panel shows the overall group comparison, the middle panel illustrates the difference scores between scanning behavior employed at the final 4 seconds before crossing and prior to these final 4 seconds, and the right panel depicts the difference scores between speed conditions. In the middle panel, a score above zero would indicate that the parameter ranking scores increases in the final 4 seconds before crossing, whereas a score below zero would indicate a decrease in the ranking scores in the final 4 seconds before crossing. In the right panel, a score above zero would indicate that the ranking score increases when cars travel 50 km/h, whereas a score below zero would indicate a decrease in the ranking scores when cars travel 50 km/h.
